# Saccadic Impairments in Patients with the Norrbottnian Form of Gaucher’s Disease Type 3

**DOI:** 10.3389/fneur.2017.00295

**Published:** 2017-06-22

**Authors:** Josefine Blume, Stanislav Beniaminov, Cecilia Kämpe Björkvall, Maciej Machaczka, Per Svenningsson

**Affiliations:** ^1^Section of Neurology, Department of Clinical Neuroscience, Center for Molecular Medicine, Karolinska Institute, Stockholm, Sweden; ^2^Hematology Center Karolinska, Department of Medicine at Huddinge, Karolinska Institutet, Karolinska University Hospital Huddinge, Stockholm, Sweden; ^3^Department of Medicine, Sunderby Regional Hospital of Norrbotten County, Luleå, Sweden

**Keywords:** Gaucher’s disease, Norrbottnian form, saccades, eye movements, antisaccades

## Abstract

**Background:**

Chronic neuronopathic Gaucher’s disease type 3 (GD3) is relatively frequent in northern Sweden. Besides multiple other neurological symptoms, horizontal gaze palsy or oculomotor apraxia is common in GD3.

**Objective:**

To characterize the saccades in patients with Norrbottnian GD3 with respect to their neurological and cognitive status using a computer-based eye-tracking technique.

**Methods:**

Horizontal and vertical reflexive saccades as well as antisaccades of nine GD3 patients [4M/5F; 41.1 ± 11.0 years; modified severity scoring tool (mSST): 9.3 ± 5.4; Montreal Cognitive Assessment (MoCA): 24.0 ± 4.2] and age-matched controls were analyzed using EyeBrain T2, a head-mounted binocular eye tracker. Systematic clinical assessment included the mSST, a valid tool for monitoring the neurological progression in GD3 and MoCA.

**Results:**

In Norrbottnian GD3 patients, gain, peak, and average velocity (107.5°/s ± 41.8 vs. 283.9°/s ± 17.0; *p* = 0.0009) of horizontal saccades were reduced compared to healthy controls (HCs). Regarding vertical saccades, only the average velocity of downward saccades was decreased (128.6°/s ± 63.4 vs. 244.1°/s ± 50.8; *p* = 0.004). Vertical and horizontal saccadic latencies were increased (294.3 ms ± 37.0 vs. 236.5 ms ± 22.4; *p* = 0.005) and the latency of horizontal reflexive saccades was correlated with the mSST score (*R*^2^ = 0.80; *p* = 0.003). The latency of antisaccades showed association to MoCA score (*R*^2^ = 0.70; *p* = 0.009). GD3 patients made more errors in the antisaccade task (41.5 ± 27.6% vs. 5.2 ± 5.8%; *p* = 0.005), and the error rate tended to correlate with the cognitive function measured in MoCA score (*p* = 0.06).

**Conclusion:**

The mean age of 41 years of our GD3 cohort reflects the increased life expectancy of patients in the Norrbottnian area compared to other GD3 cohorts. Marked impairment of horizontal saccades was evident in all patients, whereas vertical saccades showed distinct impairment of downward velocity. Latency of reflexive saccades was associated with the severity of neurological symptoms. Increased latency and error rate in the antisaccade task were linked to cognitive impairment. The assessment of saccades provides markers for neurological and neuropsychological involvement in Norrbottnian GD3.

## Introduction

Gaucher’s disease (GD) is the most common lysosomal storage disorder resulting from glucocerebrosidase (GBA) deficiency, caused by homozygote mutations in the GBA gene. Clinical manifestations are organomegaly, hematological complications, and neurological symptoms (1). The neuronopathic forms of GD emerge either acute in early childhood (GD2) or chronic Gaucher’s disease type 3 (GD3). A subtype of chronic neuronopathic phenotype is called Norrbottnian type, referring to its relatively high prevalence of 1:17,500 inhabitants of Norrbotten, a northern part of Sweden (2). The missense mutation L444P (c.1448T>C) is frequent among these patients (3). Despite the same genetic cause, the clinical course of the disease differs between individuals. The symptom burden is highly variable and can include horizontal supranuclear gaze palsy, ataxia, spastic paresis, cognitive impairment, and seizures (4). Enzyme replacement therapy is effective concerning the hematological and visceral manifestations. However, it has no favorable effect on the neurological outcome (5).

Impairment of eye movements is a common feature in lysosomal storage disorders and assessment of saccades may be a useful diagnostic tool. Different patterns of saccade impairment allow to relate pathology in the corresponding brain regions that, in turn, may allow to distinguish neurological conditions from similar symptoms with different pathophysiological substrate. For instance, patients with late-onset Tay–Sachs disease show characteristic transient decelerations and premature termination of saccades (6), whereas vertical supranuclear gaze palsy is a key clinical feature in patients with Niemann–Pick type C disease (7). In GD2 and GD3, horizontal gaze palsy or oculomotor apraxia is common and may even be the initial complain (8). Saccade analysis provides a marker for neurological involvement in GD and was already used as an outcome measurement in treatment studies (9). In a 4-year follow up of 15 GD3 patients with a median age of 15.7 years, saccade velocity was reduced and horizontal saccadic latency was increased and showed deterioration over time (10).

In this cross-sectional study, we used a computer-based eye-tracking technique to characterize the saccades in Norrbottnian GD3 patients with respect to a systematic assessment of their neurological and cognitive status. To our knowledge, this is the first study to evaluate saccades in Norrbottnian GD3 patients.

## Materials and Methods

Nine GD3 patients were recruited from among the affected patients at Norrbotten and examined at Sunderby Hospital in Luleå. Age- and sex-matched healthy controls (HCs) were examined at Karolinska University Hospital, Stockholm. The study was approved by the local research ethics committee and all participants provided written informed consent in accordance with the Declaration of Helsinki. Systematic clinical assessment was performed using the modified severity scoring tool (mSST) (11), a valid tool for monitoring neurological progression in GD3 which includes 12 items. For cognitive assessment, the Montreal Cognitive Assessment (MoCA) was used.

Eye movements were analyzed using EyeBrain T2^®^ (medical device with CE label for clinical use Class IIa, ISO 9001, ISO 13485), a head-mounted binocular eye tracker with an acquisition speed of 300 Hz. Data were acquired for both eyes by presenting stimuli on a 22 inches wide screen 60 cm away. A chin rest minimized head movement during recording. MeyeParadigm^®^ 2.1 was used to present series of stimuli and capture data. For each paradigm, a series of 12 stimuli was given after standardized verbal instructions. Paradigms included reflexive saccades with fixed target amplitudes in a horizontal (20°) and vertical (12°) step task, horizontal gap task (20°), and horizontal antisaccades (20°). The stimuli appeared outward from a central target position for a fixed period of 1,000 ms. The gap task was included to assess the rate of express saccades. For detailed information about the paradigms and parameter definition, see Data Sheet S1 in Supplementary Material.

Results are presented as mean and SD. Between group comparisons with a two-tailed significance level of 0.05 were performed using Mann–Whitney *U* test. Pearson correlation and least square regression were done to correlate quantitative variables. We refrained from multivariate regression because of small sample size.

## Results

In clinical examination, seven of the nine patients showed signs of oculomotor apraxia with delayed onset and slowness of horizontal saccades. In three individuals, distinct horizontal gaze palsy was present. In one of them, impairment of vertical eye movements was found additionally. This patient scored highest in the mSST score also and was excluded from further saccade analysis due to severe gaze palsy (patient 9). Demographic data, clinical signs, and scores are listed in Table 1.

**Table 1:** Demographic data, clinical signs, and scores.

Patient	Age	Sex	Mutation	Therapy/age	Modified severity scoring tool	Montreal cognitive assessment	Epilepsy/age of onset	Abnormal gaze	Cerebellar signs	Pyramidal signs	Extrapyramidal signs
1	50	F	L444P/L444P	ERT	5.5	26	N	Y	N	Y	N
2	43	F	L444P/L444P	Allo-BMT/9	17	19	Y/23	Y	Y	Y	Y
3	31	F	L444P/L444P	Allo-BMT/2	8.5	25	Y/16	Y	N	Y	Y
4	51	M	L444P/L444P	ERT	14	26	Y/45	Y	Y	Y	N
5	28	M	L444P/L444P	ERT	12	24	Y/17	Y	Y	N	N
6	38	F	L444P/L444P	ERT	1	26	N	N	N	N	N
7	23	M	L444P/A341T	ERT	1	30	N	N	N	Y	N
8	56	F	L444P/L444P	ERT	11.5	25	N	Y	Y	Y	N
9	50	M	L444P/L444P	ERT	13	15	N	Y	Y	Y	N
Mean	41.1	56% F	100/89%	78% ERT	9.3 ± 5.4	24.0 ± 4.2	44% Y/25.25	78% Y	56% Y	78% Y	22% Y

**Figure 1:**
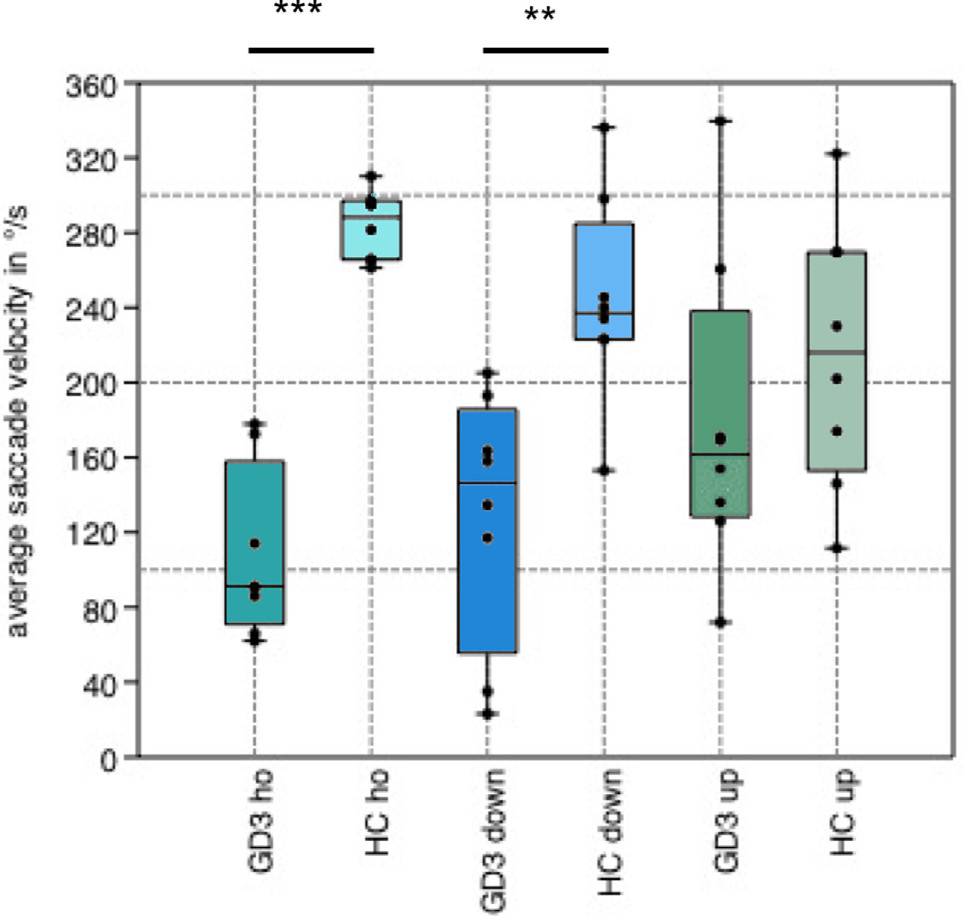
Boxplot of average saccade velocity. Boxplot showing the average saccade velocity of horizontal (ho), downward (down), and upward (up) saccades in the step paradigm. Gaucher’s disease type 3 (GD3) patients performed horizontal (*p* = 0.0009) and downward (*p* = 0.004) saccades in significantly reduced velocity compared to healthy controls (HCs), whereas there was no difference in upward saccades (*p* = 0.3).

**Figure 2:**
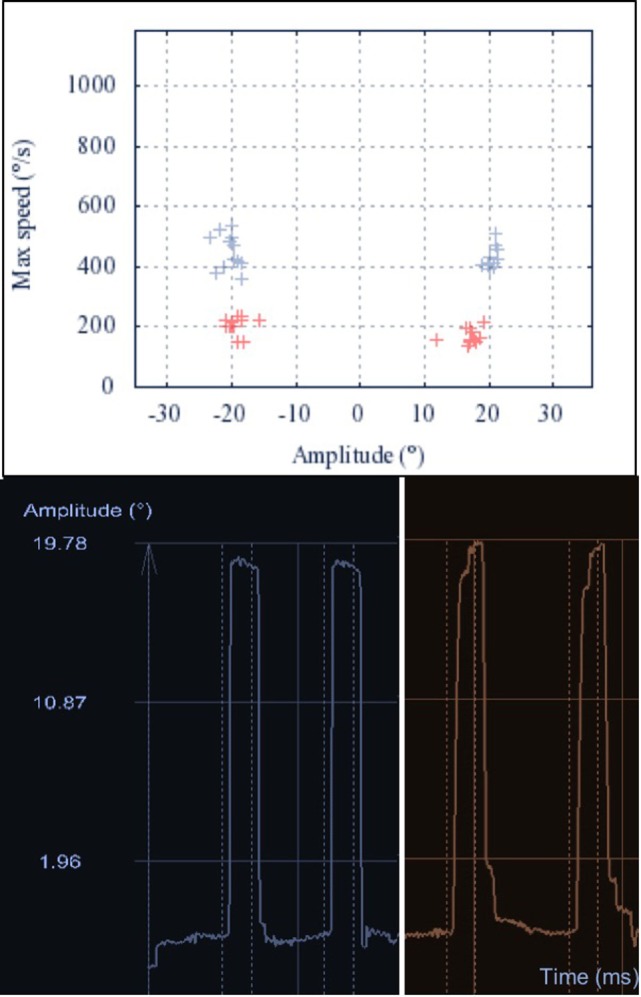
Main sequence of horizontal saccades. Upper: the main sequence illustrates the linear dependency of peak velocity and amplitude of a saccade. The main sequences of 20° horizontal saccades are shown for one Gaucher’s disease type 3 (GD3) patient (red) and one healthy control (HC) (blue). Lower: representative raw recordings of two exemplary horizontal saccades for the same GD3 patient (red, right) and HC (blue, left). The broken lines represent the appearance and disappearance of the 20° lateral stimulus. The GD3 saccades show longer duration, with decreased average velocity. Additionally, the gain of the first saccade is mildly reduced and one correction saccade is needed to reach the target.

Saccade examination using EyeBrain revealed impairment of horizontal saccades in all patients (Table 2). Average and peak velocity as well as saccadic gain of horizontal saccades were significantly decreased in GD3 patients compared to HCs (Figure 1). Figure 2 demonstrates an exemplary set of horizontal saccades of patient 5 and the age-matched HC. Since peak velocity of a saccade linearly depends on its gain for saccade amplitudes up to 20°, we illustrated the saccadic main sequence for the same patient and control additionally (Figure 2). Two severely affected individuals (patients 2 and 8) showed sustained lateral gaze with a loss of saccadic step phase and unilateral horizontal gaze palsy with markedly decreased saccade amplitudes. Solely in these two patients, the abduction–adduction ratio of average velocity was elevated over 1.0 to 1.69 and 1.6, respectively.

**Table 2:** Saccade characteristics of Gaucher’s disease type 3 (GD3) patients and controls.

	GD3 patients	Controls	*p*
Age	40.0 ± 11.2	40.0 ± 10.9	1
Sex (female/male)	5/3	5/3	1
**Latency (ms)**			
Step horizontal	294.3 ± 36.9	236.5 ± 22.4	0.005**
Step downward	293.8 ± 45.0	235.5 ± 22.4	0.004**
Step upward	301.5 ± 71.3	229.1 ± 16.7	0.002**
Gap horizontal	255.7 ± 51.3	211.4 ± 25.4	0.05
Antisaccades	271.3 ± 37.6	231.2 ± 20.9	0.01*
**Gain**			
Step horizontal	0.85 ± 0.08	0.94 ± 0.03	0.01*
Step downward	0.87 ± 0.08	0.96 ± 0.07	0.09
Step upward	0.86 ± 0.12	0.90 ± 0.03	0.7
Gap horizontal	0.81 ± 0.14	0.96 ± 0.01	0.0002***
**Average velocity (°/s)**			
Step horizontal	107.5 ± 41.8	283.9 ± 17.0	0.0009***
Step downward	128.6 ± 63.4	244.1 ± 50.8	0.004**
Step upward	178.5 ± 78.5	215.6 ± 66.1	0.3
Gap horizontal	103.5 ± 47.5	273.7 ± 28.3	0.0009***
**Peak velocity (°/s)**			
Step horizontal	226.7 ± 58.7	519.7 ± 50.5	0.0009***
Step downward	345.9 ± 195.4	455.3 ± 102.5	0.1
Step upward	393.1 ± 148.3	410.3 ± 113.5	0.9
Gap horizontal	203.0 ± 68.4	484.7 ± 66.5	0.0009***
Antisaccades error rate	41.5 ± 27.6%	5.2 ± 5.8%	0.005**
Number of express saccades	2.0 ± 2.1	1.4 ± 2.1	0.6

The significance level was established as: *p ≤ 0.05, **p ≤ 0.01, ***p ≤ 0.001. Significant results (p < 0.05) in bold.

As shown in Table 2, the gain of vertical saccades remained normal. Downward saccades were slower than upward saccades in all patients without reaching statistical significance. The average velocity of downward saccades was significantly decreased compared to HCs, whereas no difference was found in upward saccades.

**Figure 3:**
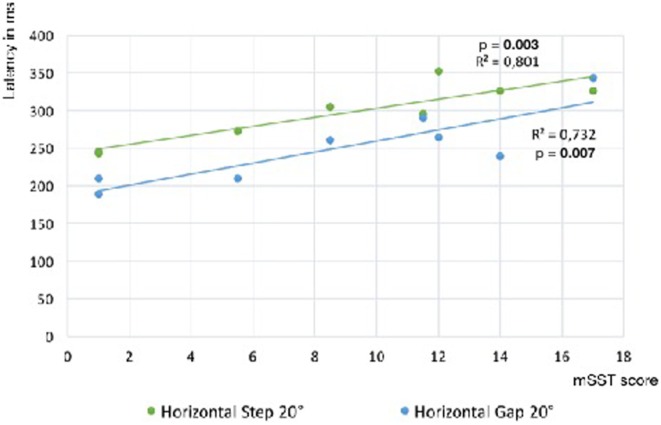
Slope of reflexive saccade latency vs. Gaucher’s disease type 3 clinical score. Linear regression slopes of saccade latency vs. modified severity scoring tool (mSST) for horizontal saccades in the step and gap paradigm. The association is significant for both paradigms (step: *R*^2^ = 0.83; *p* = 0.003; gap: *R*^2^ = 0.73; *p* = 0.007). Comparing the latencies of both paradigms, a gap effect was detectable (*p* = 0.01).

Saccade latency was prolonged compared to HCs in all paradigms, horizontal and vertical (Table 2). Latency of horizontal reflexive saccades was significantly associated with the mSST score (step: *R*^2^ = 0.80; *p* = 0.003; gap: *R*^2^ = 0.73; *p* = 0.007; Figure 3). The gap effect (12) was detectable in patients (*p* = 0.01) and controls (*p* = 0.02). We found no difference in the rates of express or anticipated saccades.

Montreal Cognitive Assessment and mSST score were associated in a bivariate linear regression model (*R*^2^ = 0.60; *p* = 0.02), while both showed no association to age (*p* = 0.5 and *p* = 0.2, respectively). The antisaccade latency was associated with MoCA score (*R*^2^ = 0.70; *p* = 0.009), but not to age (*p* = 0.5). GD3 patients made more errors in the antisaccade task than HCs. The antisaccade error rate showed significant correlation to age (*R*^2^ = 0.54; *p* = 0.04) and tended to correlate with mSST scores (*R*^2^ = 0.50; *p* = 0.052) and to MoCA score (*R*^2^ = 0.47; *p* = 0.06).

## Discussion

In this paper, we describe the characteristics of saccades in nine patients with Norrbottnian type GD3 with respect to their neurological and cognitive status for the first time. Impairment of horizontal gaze is the most frequent neurological feature in GD3 and, indeed, was found in all patients when assessed using a computer-based eye-tracking technique. The mean age of 41 years in our GD3 cohort reflects the increased life expectancy and milder course of the disease in patients from the Norrbottnian area compared to other GD3 cohorts.

In Norrbottnian GD3 patients, gain and velocity were clearly abnormal in horizontal saccades compared to healthy individuals. Regarding vertical saccades, only average velocity of downward saccades was decreased while upward saccades appeared normal, besides prolonged latency. The distinct impairment of downward saccades is a feature in other neurodegenerative diseases with predominant impairment of vertical gaze like Niemann–Pick type C (13) and progressive supranuclear palsy as well. However, the pathophysiological mechanisms underlying the differential effects on midbrain pathways for downward and upward saccade generation in these diseases are not fully understood. Reduced velocity of horizontal saccades is caused by affection of the premotor burst neurons in the ipsilateral paramedian pontine reticular formation, whereas slowing of vertical saccades indicates a subsequent involvement of the rostral interstitial medial longitudinal fascicle (14), which may be caused by spreading of GD3 pathology in advanced stages of the disease. Here, additional involvement of omnipause neurons in the raphe interpositus nucleus may lead to further slowing of both horizontal and vertical saccades.

Two patients with severe neurological symptoms showed sustained lateral gaze and an increased abduction/adduction velocity ratio, which may indicate additional involvement of internuclear neurons or the medial longitudinal fasciculus that link the ipsilateral abducens nucleus to the contralateral oculomotorius nucleus. Horizontal saccadic hypometria may be caused by spreading of GD3 pathology to neuronal integrator cells in cerebellar dorsal vermis or nucleus prepositus hypoglossi (14). The exact evolution of neuropathological changes in GD3 in relation to the clinical course is still unknown. Brains of advanced stage GD3 patients showed widespread perivascular Gaucher cells as well as gliosis and neuronal cell loss in brainstem and cerebellum (15). However, no clear accentuation of neuropathological changes was found in the pons.

The delayed initiation of saccades, measured as prolonged latency, led to the term oculomotor apraxia in GD (6). Horizontal saccadic latency was associated with the severity of neurological symptoms measured in mSST in our cohort. An increased latency reflects alterations in the oculomotor processing above the brainstem level, e.g., cortical dysfunction affecting the frontal or parietal eye field (14). The severity of neurological involvement, especially the presence of epilepsy, may be caused by more wide-ranging Gaucher pathology in supratentorial areas. A study using diffusion-weighted magnetic resonance imaging in 13 infantile neuronopathic GD2 patients demonstrated reduced diffusion coefficient (ADC) values in cortical temporal, cortical and subcortical frontal regions, corticospinal tract, cerebellum, and midbrain compared to HCs (16), suggesting extensive alterations of tissue integrity in these regions. In our study, four of the nine patients suffered from epilepsy, mostly focal dyscognitive seizures beginning in adolescent or in adulthood, but no progressive myoclonic epilepsy. Patients with epilepsy showed significantly longer latencies of horizontal saccades, but no difference in their velocity compared to patients without epilepsy. However, an influence of the antiepileptic treatment on saccade performance is possible. On the other hand, patients with epilepsy were more affected by GD3 in several ways, showed more ataxia, extrapyramidal signs, and spasticity, which suggest more severe involvement of higher brain areas. Furthermore, the differences to HCs in all saccade parameters remained significant when only GD3 patients without epilepsy were included in the analysis.

The performance in the antisaccade task is associated with functional and imaging markers of executive function in healthy individuals and several neurodegenerative diseases (17). In our GD3 cohort, antisaccade errors tended to be associated with the cognitive function measured in MoCA. Additionally, the antisaccade latency correlated with MoCA, but not to mSST score or age and may be an age-independent marker for supratentorial involvement in GD3. As antisaccades are known to reflect frontal-based functions, a more focused assessment of executive tasks in a larger sample of patients could be helpful to prove our findings.

In summary, our results strengthen the findings of former studies of eye movements in GD (8) and provide additional evidence that saccadic impairment reflects neurological and neuropsychological involvement in GD3, including the Norrbottnian type.

## Ethics Statement

All subjects gave written informed consent in accordance with the Declaration of Helsinki. The protocol was approved by the local ethics committee of the Karolinska University Hospital, Stockholm.

## Author Contributions

Conception of the study and substantial manuscript drafting: JB and PS. Acquisition of data: JB, PS, CB, MM, and SB. Analysis of data: JB, PS, and SB.

## Conflict of Interest Statement

The authors declare that the research was conducted in the absence of any commercial or financial relationships that could be construed as a potential conflict of interest.
