# Supplementary material for: Saccadic Impairments in Patients with the Norrbottnian Form of Gaucher’s Disease Type 3
Source: Front Neurol. 2017 Jun 22;8:295. doi: 10.3389/fneur.2017.00295 (PMC5479920; doi:10.3389/fneur.2017.00295)
Supplement: Supplementary file 1 [file Data_Sheet_1.DOCX]

**Supplementary material 1: eye tracking paradigm and parameter definition**

Eye movements were analyzed using EyeBrain T2® (Medical device with CE label for clinical use Class IIa, ISO 9001, ISO 13485), a head-mounted binocular eye-tracker with acquisition speed at 300Hz. The data was acquired for both eyes by presenting stimuli on a 22 inches wide screen 60 cm away, a chin rest minimized head movement during recording. MeyeParadigm® 2.1 was used to present series of stimuli and capture data.

The subjects completed a set of reflexive saccades to calibrate the eye tracker to screen space before each assessment. For each paradigm, a series of stimuli was given for 60 seconds after standardized verbal instructions. Paradigms included:

Gap horizontal: The patient was instructed to look at the luminous target, located in either a central or lateral position. The central fixation target was shown for 2500 ms to 3500 ms. When it disappeared, a black screen was shown for 200 ms. Then, a lateral fixation target appeared at 20° on the right or left side. This target remained visible for 1000 ms. Finally, the lateral target disappeared and a new central fixation target was shown.

Step horizontal: The patient was instructed to look at the luminous target, located in either a central or lateral position. The central fixation target was shown for 2500 to 3500 ms. As soon as it disappeared a lateral fixation target simultaneously appeared 20° on the right or left side. This target remained visible for 1000 ms. Finally, the lateral target disappeared and a new central fixation target was shown.

Step vertical: The patient is instructed to follow a luminous target located in a central position or in a peripheral position (up or down). The central fixation target was shown for 2500 to 3500 ms. As soon as it disappeared, a peripheral fixation target appeared, located 12° up or down. This target remained visible for 1000 ms. Finally, the peripheral target disappeared and a new central fixation target was shown.

Antisaccades: The central fixation target was shown for 3500 to 5500 ms. When it disappeared, a black screen was shown for 200 ms. Then, a lateral target appeared 20° to right or left. This target remains visible for 1000 ms. After a central fixation period when a lateral target appeared, the patient had to make a saccade in the opposite direction to that of the target.

Measured parameters were defined as:

Saccadic latency: the time between presentation of a stimulus and triggering of the saccade.

Saccadic velocity: the velocity between the start and the end of the saccade and including mean velocity, peak velocity and skewness.

Saccadic gain: angular position of the main saccade's fixation plateau with respect to the angular position of final saccade’s plateau.

Antisaccade errors: number of reflexive saccades to the peripheral target instead of the opposite direction during the antisaccades task.

Anticipated and express saccades: number of saccades with a latency shorter than 70ms, respectively between 70ms and 135ms.
